# Pertussis resurgence in Toronto, Canada: a population-based study including test-incidence feedback modeling

**DOI:** 10.1186/1471-2458-11-694

**Published:** 2011-09-07

**Authors:** David N Fisman, Patrick Tang, Tanya Hauck, Susan Richardson, Steven J Drews, Donald E Low, Frances Jamieson

**Affiliations:** 1 Dalla Lana School of Public Health, University of Toronto, 155 College Street, Toronto, M5T 3M7, Canada; 2 Department of Health Policy, Evaluation and Management, University of Toronto, 155 College Street, Toronto, M5T 3M7, Canada; 3 Department of Medicine, University of Toronto, 1 Kings College Circle, Toronto, M5S 1A8, Canada; 4 Public Health Laboratory--Toronto, Ontario Agency for Health Protection and Promotion, 81 Resources Road, Toronto, M9P 3V6, Canada; 5 Department of Laboratory Medicine and Pathobiology, University of Toronto, 1 Kings College Circle, Toronto, M5S 1A8, Canada; 6 Department of Microbiology, Hospital for Sick Children, 555 University Avenue, Toronto M5G 1X5, Canada; 7 Alberta Provincial Public Health Laboratory, 3030 Hospital Drive Northwest, Calgary, T2N 4W4, Canada; 8 Department of Microbiology, Mount Sinai Hospital, 600 University Avenue, Toronto, M5G 1X5, Canada

## Abstract

**Background:**

Pertussis continues to challenge medical professionals; recently described increases in incidence may be due to age-cohort effects, vaccine effectiveness, or changes in testing patterns. Toronto, Canada has recently experienced increases in pertussis incidence, and provides an ideal jurisdiction for evaluating pertussis epidemiology due to centralized testing. We evaluated pertussis trends in Toronto using all available specimen data, which allowed us to control for changing testing patterns and practices.

**Methods:**

Data included all pertussis culture and PCR test records for Greater Toronto from 1993 to 2007. We estimated incidence trends using Poisson regression models; complex relationships between disease incidence and test submission were explored with vector autoregressive models.

**Results:**

From 1993 to 2007, 26988 specimens were submitted for testing; 2545 (9.4%) were positive. Pertussis incidence was 2 per 100,000 from 1993 to 2004 and increased to 10 per 100,000 from 2005-2007, with a concomitant 6-fold surge in test specimen submissions after the introduction of a new, more sensitive PCR assay. The relative change in incidence was less marked after adjustment for testing volumes. Bidirectional feedbacks between test positivity and test submissions were identified.

**Conclusions:**

Toronto's recent surge in pertussis reflects a true increase in local disease activity; the apparent size of the outbreak has likely been magnified by increasing use of pertussis testing by clinicians, and by improved test sensitivity since 2005. These findings may be applicable to changes in pertussis epidemiology that have been noted elsewhere in North America.

## Background

Pertussis is a highly contagious respiratory tract infection caused by the gram negative bacterium *Bordetella pertussis *or less commonly by *B. parapertussis *[1]. The disease is classically characterized by three stages [2]: (i) a nonspecific catarrhal stage; (ii) a subsequent spasmodic stage involves the characteristic paroxysmal cough with inspiratory whoop,[3] and lasting two to eight weeks; and (iii) a convalescent phase [3]. The pathogenesis of the disease is not fully understood but involves both direct toxic effects of bacterial endo- and exotoxins, and also indirect effects of toxins on the host immune response, including a reduction in lymphocyte circulation [1,4,5]. While children and adults of any age may develop pertussis, severe sequelae (including encephalopathy and pneumonia) are most common in infants aged < 6 months [6-8]. The incidence of pertussis has decreased dramatically in wealthy countries since the implementation of widespread vaccination programs (e.g., from ~170/100 000 in the 1930s to fewer than 20/100 000 in the 1970s in Canada)[9] pertussis is still an important source of mortality worldwide. The disease remains one of the leading causes of infant mortality,[6-8] and causes approximately 300 000 deaths in 50 million cases per year [7,10].

In high income countries, elimination of pertussis has not occurred despite immunization, and apparent disease incidence has increased in recent decades [3,6,11]. Recent outbreaks, some with infant deaths, in Nottingham and Derby (England) [12], California [13], Ireland [14] and New South Wales, Australia [15] have been a source of considerable concern. It has been suggested, however, that increasingly sensitive PCR methods for the diagnosis of pertussis may have contributed to apparent, rather than real, increases in disease incidence [7,9,16-18]. Other factors invoked to explain pertussis increases in countries with high rates of immunization include genetic changes in the pathogen, [19,20] waning immunity following both vaccination and infection,[1,3,7] reduced potency of vaccines following the switch to an acellular vaccine,[21,22] more widespread laboratory testing due to increased awareness of the disease in adults,[22] and lack of immunity or waning immunity in specific age cohorts [23]. The role played by young adolescents and adults in disease spread,[24] and the recognition of a gradual loss in immunity after natural infection or vaccination, have led several countries to advocate booster dosing of pertussis vaccine for young teens and adults [16,25-27].

Surveillance of pertussis is complicated by differing manifestations of the disease in infants, adolescents and adults [28], with the disease presenting as apnea or poor feeding in infants [2,12], whereas adults and adolescents often present only with prolonged cough [11], or minimal symptomatology [6]. Nasopharyngeal swab culture is the historical gold-standard diagnostic modality for pertussis, but is sensitive only early in the infection, with a sensitivity < 50% after three weeks of illness or cough [8]. Serology is even less sensitive but can be used to diagnose pertussis late in the course of infection [7]. In recent years, the sensitivity and rapidity of polymerase chain reaction (PCR) have brought this testing modality into widespread use, even if testing is not well-standardized between laboratories [8,16-18]. The World Health Organization's (WHO) most recent guidelines define a pertussis case as clinically confirmed (without laboratory confirmation) or laboratory confirmed (and meeting the clinical case definition)[10]. Prior to 2008, Canadian case definitions permitted the classification of a case of pertussis as "confirmed" based on a positive laboratory test (including PCR) alone [29,30].

The Greater Toronto Area (GTA) is Canada's largest metropolitan area. All pertussis testing in the GTA is performed in only two laboratories (the Public Health Laboratory--Toronto (PHLT) and the laboratory of the Hospital for Sick Children (HSC), which share strong historical linkages, making it possible to evaluate not only case counts (according to pre-2008 Canadian case definitions) via laboratory data, but also to evaluate the impact that testing volume and testing practice may have had on measured disease epidemiology. The region experienced a documented outbreak of pertussis in 2005-2006 [7,9,16], and experienced a prolonged increase in pertussis incidence in association with the introduction of novel testing methodologies. Our objectives were to evaluate changes in pertussis epidemiology in this large urban setting and to define the relative contributions to disease incidence that may be attributable to laboratory testing submission volumes, changing testing technologies and underlying disease epidemiology.

## Methods

### Demographics and Laboratory Characteristics

The Greater Toronto Area (GTA) is the most populous metropolitan area in Ontario, with a population of 5,113,149 in 2006 over 7,125 km^2^[31]. The GTA is located in the southern part of Ontario and is comprised of five administrative regions (Toronto, York, Halton, Peel and Durham), all of which have separate public health units charged with disease prevention and control activities. Pertussis is a notifiable infectious disease in Ontario and must be reported to regional public health agencies if compatible with the national case definition: during the period reported here, a confirmed case was one with either isolation of *B. pertussis *or a positive PCR assay for *B. pertussis*, regardless of symptomatology [29]. Classical symptoms of pertussis (paroxysmal cough, post-tussive vomiting or apnea, and inspiratory whoop) are considered confirmatory in the absence of laboratory testing of individuals with known epidemiological links to another confirmed case [29]. Typical case management strategies employed by public health authorities include evaluation of vaccine status, with provision of catch-up or booster vaccination as appropriate, and provision of post-exposure antimicrobial therapy to close contacts of cases [32].

GTA pertussis testing is conducted by two laboratories: the Public Health Laboratory--Toronto (PHLT) and the Hospital for Sick Children (HSC). The PHLT provides primary diagnostic services for hospitals and healthcare practices in the GTA, as well as confirmatory testing and reference laboratory services for the entire province. HSC's laboratory performs testing principally on children from the hospital's inpatient and outpatient areas, and the hospital's emergency room. Data included here cover all pertussis culture and PCR tests conducted at these two sites from 1993 to 2007. Serological data are not available. Prior to 1999, only culture isolation of *B. pertussis *was available.

### Evolution of Testing Procedures

Data on respiratory specimen testing at the PHLT were available from January 1993 to January 2007. Since January 1999, the PHLT has performed parallel culture and PCR on all respiratory specimens submitted for pertussis testing, with culture and identification of *B. pertussis *performed as described elsewhere [33]. The PCR assay introduced at the PHLT in 1999 was a qualitative assay for the presence of the IS481 gene, an insertion sequence that appears in multiple copies in the pertussis genome, and which is not present in several other *Bordetella *species of public health importance (e.g., *B. parapertussis*, *B. bronchoseptica*)[16]. The introduction of PCR in January 1999 was associated with a change in specimen collection procedures for pertussis testing, with the collection media changed from nutrient-rich, antibiotic impregnated Regan-Lowe medium, to phosphate-buffered-saline (PBS) medium. In May 2005, the PHLT introduced a real-time, quantitative PCR assay incorporating a fluorescent probe for enhanced assay sensitivity. Data on respiratory specimens submitted to the HSC laboratory were available from October 1999 to January 2007; a qualitative IS481-directed PCR was used on all specimens submitted at HSC during this time period, and culture was not performed.

### Statistical Analysis

Age- and sex-specific population estimates for the Toronto Census Metropolitan Area were obtained from the 1996, 2001, and 2006 Canadian census [31]; data for inter-census years were estimated through linear interpolation and extrapolation. We estimated crude and adjusted incidence of pertussis testing and disease occurrence in the Greater Toronto Area using specimens linked by postal code forward sortation area (FSA, the first three alphanumeric characters of the six character Canadian postal code) to the GTA, or which were identified as having been obtained in one of the five jurisdictions comprising the GTA (when FSA was absent). A small number of test specimens processed by the HSC laboratory were obtained from individuals with home FSA outside the GTA. HSC is a major pediatric tertiary/quaternary care center in Ontario, and consequently provides care to many patients who reside outside the GTA. When non-GTA specimens were obtained from individuals identified as hospital inpatients, these test specimens were included in counts for the Toronto health region. When non-GTA specimens processed by HSC were identified as being from outpatients, and when specimens were identified as having been provided by outside laboratories for reference testing, specimens were excluded from further analysis.

Risk of pertussis by age, gender, season, and time period were evaluated using census-derived person-time denominators, with differences in risk evaluated through calculation of risk differences and 95% confidence limits. We compared age distributions for cases by time period (prior to 1999, from 1999 to May 2005, and after May 2005) using the Wilcoxon rank-sum test. Data were missing for subject gender (5.9%) and age (3.0%). In our primary analysis, individuals missing values for age were replaced using random age values drawn from a log-normal distribution with a mean and standard deviation identical to that of known ages. Missing gender was replaced randomly in proportion to known gender distributions. Sensitivity analyses were also performed, with records with missing data excluded from analyses.

Specimen factors associated with test positivity, and with culture or PCR positivity, using categorical methods, as well as univariable and multivariable logistic regression models that included age and gender of the specimen source person, laboratory performing the tests, and year and season of submission (modeled using "fast Fourier transforms" (FFT) [34] that provided an underlying sinusoidal seasonal baseline).

Temporal trends in pertussis occurrence for the population as a whole, and for individual demographic groups were explored using multivariable Poisson regression models that included both seasonal smoothers (FFT as described above) and also oscillatory FFT with 6-year periodicity, to account for the previously described multi-year periodicity of pertussis [35]. The assumption of Poisson-distribution of errors was evaluated with the deviance statistic [36] and found to be appropriate (*P *= 1.00). The impact of changing testing procedures on test positivity was evaluated by introducing step functions at time points where HSC data were added to the time series, and where test procedures were modified (i.e., with the introduction of PCR by the PHLT and HSC laboratories, and with the modification of PCR testing procedures by the PHLT). Typical time series models of public health surveillance data are not able to account for the impact of the volume of test submission on apparent disease incidence (i.e., lack so-called "testing denominators"); test submission volumes in multivariable models were included, to evaluate the significance of their contribution to model fit using the log-likelihood test [37]. We performed exploratory analyses in which we evaluated the differential effects of test volume on apparent pertussis incidence both by incorporating multiplicative interaction terms into models, and by performing restriction analyses in which testing effects were evaluated in models restricted to a single testing period (i.e., prior to 1999, from January 1999 to April 2005, and from May 2005). We evaluated differences in testing-adjusted and unadjusted covariates using the meta-analytic Q-statistic [38], and evaluated heterogeneity in testing effects by period using the Wald chi-squared test [38]. Model choice was guided by balancing model fit against parsimony through minimization of Akaike's information criterion [39].

We hypothesized that the relationship between test submission and test positivity would be complex and bidirectional, and assessed the relationship between submission and positivity through construction of vector autoregressive models, with evidence of causality sought through the application of Granger's test [40]. Briefly, this statistical test (widely applied in econometrics) seeks to identify cause and effect relationships by comparing model prediction when dependent variables and independent variables (at variable lags) are reversed [40]. Evidence of stationarity of time series using the Dickey-Fuller unit root test [41] was assessed.

All data analysis was performed with Stata version 11 (StataCorp, College Station, TX). The study was approved by the Research Ethics Board of the University of Toronto.

## Results

### Epidemiological Profile of Pertussis in the Greater Toronto Area

**Figure 1:**
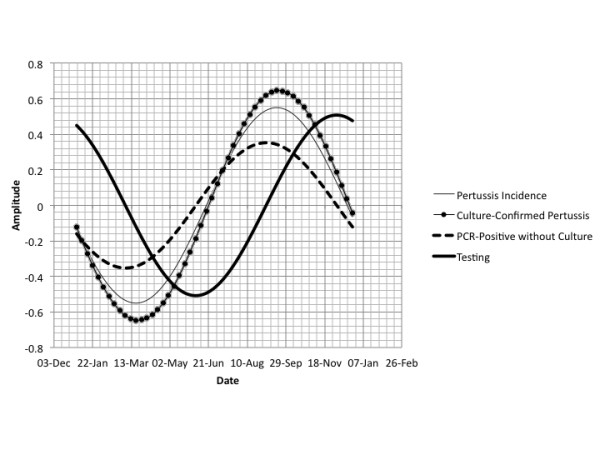
**Comparative Best-Fit Seasonal Waveforms for Pertussis and Pertussis Testing**. Pertussis incidence (thin solid curve), pertussis culture positivity (dotted curve), and pertussis PCR positivity (dashed curve) all display significant autumn seasonality; testing volumes (dark solid curve) are also distinctly seasonal but surge in winter.

In total, 26,988 test records were evaluated, of which 2545 (9.4%) tested positive for pertussis. PHLT provided 82.1% of the records and 17.9% came from HSC. Females accounted for 50.7% of samples and most were infants, children or adolescents, with 53.4% under 5 and 78.4% under the age of 15 years. There was no clinically significant change in proportions tested by age or gender when observations with missing age and gender classification were excluded from analyses (data not shown). The average risk of pertussis during the period under study was 3.68 cases per 100,000 person-years (95% CI 3.54 to 3.82). The incidence of identified disease declined sharply with age, and incidence was highest in autumn and winter months. The age distribution of pertussis cases varied significantly across the three testing periods (P < 0.001 for all two-way comparisons by Wilcoxon rank-sum test) but there were no consistent patterns observed in changing age composition of cases over time. Crude pertussis incidence changed minimally with the introduction of diagnostic PCR in 1999 (risk difference 0 per 100,000, 95% CI -0.01 to 0.02). In contrast, the introduction of a novel, highly sensitive PCR assay in May 2005 was associated with a marked increase in pertussis incidence (risk difference 8.09 per 100,000, 95% CI 7.73 to 8.47) (Figure 1 and Table 1).

**Table 1:** Crude and Stratum-Specific Rates of Laboratory-Confirmed Pertussis in Greater Toronto Area, 1993-2007

Group	Rate per 100,000 Person-Years (95% Confidence Interval)			
Overall	3.68	3.54	to	3.82
Age				
0-4	31.39	29.79	to	33.08
5-9	9.46	8.60	to	10.40
10-14	7.43	6.68	to	8.26
15-19	2.05	1.67	to	2.51
20+	0.57	0.51	to	0.64
Gender				
Male	3.58	3.39	to	3.79
Female	3.77	3.57	to	3.98
Time Period				
Prior to Introduction of PCR (January 1993-December 1998)	2.06	1.89	to	2.24
Initial Introduction of PCR Testing (January 1999-April 2005)	2.06	1.91	to	2.23
Introduction of Highly Sensitive PCR (May 2005-)	10.15	9.64	to	10.70

In multivariable Poisson models, we identified significant seasonal oscillation in incidence, and also significant oscillation with 6-year periodicity. Differences in disease incidence by age group were statistically significant in multivariable Poisson models that adjusted for such temporal trends; in adjusted models, the risk of pertussis was significantly greater in females than males. There was no net change in disease incidence after the initial introduction of PCR in January 1999, and a marked increase in incidence coincident with the introduction of a highly sensitive pertussis assay in May 2005. Test volume increased sharply after May 2005 as well; after adjustment for test volume, the relative increase in pertussis after May 2005 was diminished (*P *for heterogeneity < 0.001) though it remained clinically and statistically significant (Table 2).

**Table 2:** Poisson Regression on Demographic Risks, Temporal Trends, and Testing Effects Associated with Reported Pertussis Risk in the Greater Toronto Area, Canada

	Exclude Testing Volume		Include Testing Volume	
**Variable**	**IRR (95% C.I.)**	**P-value**	**IRR (95% C.I.)**	**P-value**

Temporal Trends*				
Seasonal Oscillation	---	0.04	---	< 0.001
Oscillation, 6-year Period	---	< 0.001	---	< 0.001
Demographic Characteristics				
Female Gender	1.15 (1.06 to 1.24)	< 0.001	1.14 (1.05 to 1.23)	< 0.001
Age				
0 to 4 years	57.76 (50.91 to 65.53)	< 0.001	38.59 (33.84 to 44.00)	< 0.001
5 to 9 years	17.16 (14.78 to 19.93)	< 0.001	17.34 (14.94 to 20.14)	< 0.001
10 to 14 years	13.14 (11.24 to 15.36)	< 0.001	14.81 (12.66 to 17.32)	< 0.001
15 to 19 years	3.57 (2.82 to 4.52)	< 0.001	4.30 (3.40 to 5.44)	< 0.001
20 and over	1 (referent)	---	1 (referent)	---
Testing Practices				
Introduction of Novel, Highly Sensitive PCR (May 2005)	3.75 (3.44 to 4.09)	< 0.001	2.31 (2.09 to 2.55)	< 0.001
Test Submission Volume (per 10 additional test specimens)	---	---	1.57 (1.53 to 1.61)	< 0.001
Pseudo-R^2^	0.43		0.47	
Akaike's Information Criterion	11325		10421	

**NOTE: **IRR, incidence rate ratio; CI, confidence interval.

* P-values for oscillation based on sine and cosine terms for oscillation at yearly and 6-yearly intervals.

The impact of test submission volume on apparent pertussis incidence was diminished after May 2005 as opposed to before May 2005 (IRR per 10 specimens submitted 1.30, 95% CI 1.26 to 1.35 vs. IRR 7.93, 95% CI 6.80 to 9.26, *P *for heterogeneity < 0.001). When this time period was excluded there was no heterogeneity in the effect of test submissions before as opposed to after the introduction of PCR in January 1999 (IRR per 10 specimens submitted 9.30, 95% CI 7.74 to 11.18 vs. IRR 4.59, 95% CI 3.35 to 6.27, *P *= 0.14).

### Patterns in Laboratory Testing in the Greater Toronto Area

Seasonal oscillation in pertussis incidence using fast Fourier transforms were incorporated into Poisson models for the time period prior to May 2005. Pertussis incidence, incidence of culture-confirmed pertussis, incidence of pertussis without culture confirmation, and pertussis test submissions all displayed statistically significant annual seasonality (P for oscillation < 0.001 for all evaluations). However, while test positivity for both culture- and PCR-positive pertussis displayed autumn seasonality (peak occurrence weeks 36 to 38), pertussis testing peaked in winter (week 49) (Figure 1**)**. Heterogeneity in phase terms was assessed using the meta-analytic Q-statistic and found significant heterogeneity in waves (Q-statistic 778.2 on 3 *d.f*., P < 0.001); however, the wave forms for seasonality of test-positivity were homogeneous (Q-statistic 2.4 on 3 *d.f*., P < 0.31).

**Figure 2:**
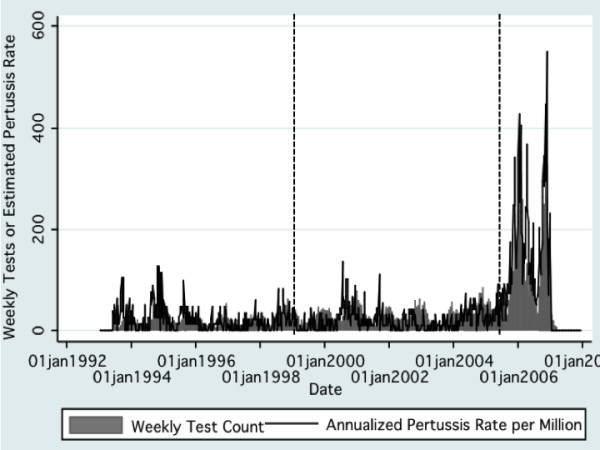
**Temporal Trends in Pertussis Testing and Estimated Crude Pertussis Incidence, Greater Toronto Area, Canada, 1993-2007**. Cumulative weekly tests are presented as gray bars; annualized pertussis rates are presented as rates per million rather than per 100,000 (as in text) to maintain comparability of scales. Dashed lines represent initial introduction of PCR testing (January 1999) and introduction of more sensitive PCR assay (May 2005). It can be seen that there is a strong correlation between specimen submission and estimated pertussis incidence.

**Figure 3:**
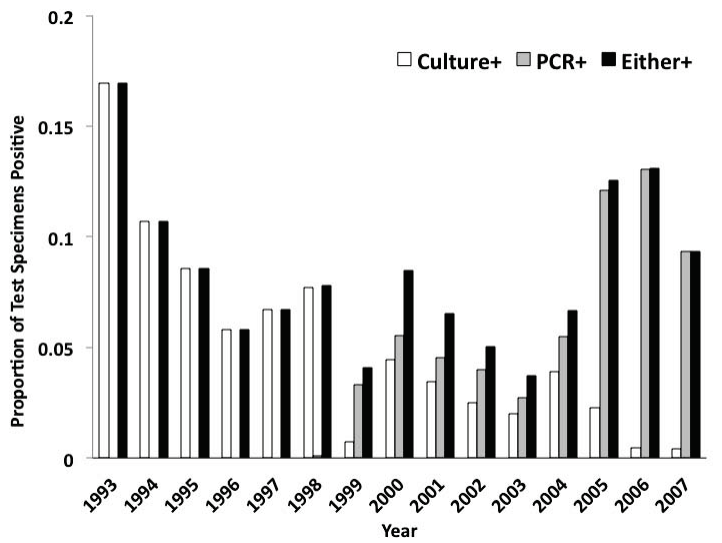
**Proportion of Test Submissions Positive by Year**. The fraction of tests positive by culture (white bars) has declined over time, but the fraction positive by PCR (gray bars) has increased since 2000, with a concomitant rebound in the overall proportion of test specimens testing positive.

Submitted specimen volumes increased over the period of observation: the increase was gradual prior to 1999 (IRR per year 1.06, 95% CI 1.05 to 1.07), and accelerated with the introduction of PCR (IRR 1.11, 95% CI 1.05 to 1.18). There was a sharp increase in test submission after May 2005 (IRR 2.72, 95% CI 2.61 to 2.83) (Figure 2). During the period under study, 2545 tests (9.4%) were positive by either culture or PCR (Figure 3). Of the positive tests, 63.9% were positive by PCR but negative by culture, 9.2% were positive by both assays, and the remaining 26.9% were culture positive without PCR positivity (most of this latter group had been evaluated at the PHLT prior to the introduction of PCR).

In multivariable logistic regression models constructed using 1858 specimens positive for pertussis at the PHLT from 1999 to 2007 (i.e., in a setting where both PCR and culture were performed in tandem on submitted specimens), the likelihood of PCR-positive pertussis without culture confirmation increased over time, and with the introduction of the novel PCR assay in May 2005, and was elevated in younger age groups, but did not vary by gender (P = 0.32) or season (P for seasonal oscillation = 0.06) though the latter was retained in the model to adjust for residual confounding (Table 3).

**Table 3:** Clinical, Temporal and Testing Characteristics Associated with PCR-Positive, Culture-Negative Specimens (N = 1858), 1999-2005

Specimen Characteristic	Odds Ratio	95%		CI	P-value
Age 0-4	2.61	1.71	To	3.98	< 0.001
Age 5-9	1.73	1.02	To	2.93	0.04
Age 10-14	0.41	0.25	To	0.65	< 0.001
Age 15-19	0.41	0.20	To	0.82	0.01
Age > 20 (referent)	1.00	---	---	---	---
Time (years)	1.13	1.03	To	1.25	0.01
Introduction of Novel PCR (May 2005)	16.35	10.19	To	26.23	< 0.001

NOTE: Model also included sine and cosine terms to adjust for residual confounding by season (see text). CI, confidence interval.

We used vector autoregressive models to evaluate the temporal links between pertussis test positivity and test submissions. We found strong statistical evidence for stationarity of both time series for tests (P < 0.001) and positive tests (P = 0.003). In vector autoregressive models with 4-week lags test positivity predicted subsequent test submissions, and test submissions predicted subsequent test positivity (Table 4), with strong evidence for Granger causality over the time period under study. However, when we restricted our analysis to the period prior to the introduction of the novel PCR assay (prior to May 2005), test positivity was found to be Granger causal of subsequent test submission, but not vice versa. During the period from May 2005 to December 2007, test submission and test positivity were Granger causal of one another.

**Table 4:** Vector Autoregressive Model Evaluating Prediction of Pertussis Test Positivity by Lagged Pertussis Positivity and Test Submissions

Independent Variable and Lag	Entire Study Period	(1993-2007)	Prior to Novel PCR	(1993 to April 2005)	Novel PCR	(May 2005 to December 2007)
	Coefficient (95% CI)	P-value	Coefficient (95% CI)	P-value	Coefficient (95% CI)	P-value
Test Positivity		< 0.001*		0.55*		0.003*
1 week lag	0.49 (0.41 to 0.58)	< 0.001	0.20 (0.12 to 0.28)	< 0.001	0.56 (0.34 to 0.79)	< 0.001
2 week lag	0.42 (0.33 to 0.51)	< 0.001	0.22 (0.14 to 0.30)	< 0.001	0.55 (0.31 to 0.79)	< 0.001
3 week lag	0.16 (0.07 to 0.25)	< 0.001	0.18 (0.10 to 0.27)	< 0.001	0.10 (-0.14 to 0.34)	0.42
4 week lag	-0.22 (-0.31 to -0.14)	< 0.001	0.07 (-0.01 to 0.16)	0.07	-0.42 (-0.64 to -0.20)	< 0.001
Test Volume						
1 week lag	0.02 (0 to 0.04)	0.05	0.01 (-0.01 TO 0.02)	0.52	0.02 (-0.03 to 0.07)	0.41
2 week lag	-0.06 (-0.08 to -0.04)	< 0.001	-0.02 (-0.04 TO 0.01)	0.08	-0.09 (-0.15 to -0.03)	< 0.001
3 week lag	0.03 (0.01 to 0.06)	< 0.001	0.01 (-0.01 TO 0.03)	0.48	0.07 (0.01 to 0.13)	0.03
4 week lag	0.01 (0 to 0.03)	0.11	0.00 (-0.01 TO 0.02)	0.68	0.02 (-0.02 to 0.07)	0.27
						
Test Positivity		< 0.001*		< 0.001*		< 0.001*
1 week lag	1.41 (1.03 to 1.79)	< 0.001	0.44 (0.08 TO 0.80)	0.02	1.81 (0.78 to 2.83)	< 0.001
2 week lag	0.61 (0.21 to 1.01)	< 0.001	0.57 (0.21 TO 0.93)	< 0.001	0.94 (-0.15 to 2.02)	0.09
3 week lag	-0.05 _0.45 to 0.36)	0.83	0.17 (-0.20 TO 0.53)	0.37	-0.14 (-1.24 to 0.96)	0.81
4 week lag	-1.04 (-1.42 to -0.67)	< 0.001	-0.20 (-0.56 to 0.16)	0.28	-1.30 (-2.28 to -0.32)	0.01
Test Volume						
1 week lag	0.66 (0.58 to 0.74)	< 0.001	0.49 (0.41 to 0.57)	< 0.001	0.67 (0.45 to 0.90)	< 0.001
2 week lag	0.01 (-0.08 to 0.11)	0.77	0.22 (0.13 to 0.31)	< 0.001	-0.15 (-0.41 to 0.12)	0.29
3 week lag	0.25 (0.15 to 0.35)	< 0.001	0.12 (0.04 to 0.21)	0.01	0.39 (0.12 to 0.66)	0.01
4 week lag	-0.08 (-0.16 to 0)	0.04	0.05 (-0.03 to 0.12)	0.26	-0.11 (-0.30 to 0.09)	0.28

NOTE: CI, confidence interval; * signifies P-value from Granger causality test.

## Discussion

Despite a greater than 10-fold reduction in the burden of pertussis-related mortality and morbidity in high- and middle-income countries with the introduction of immunization, this disease continues to challenge clinicians and public health policymakers [11,42]. These challenges are rooted in fundamental questions regarding the epidemiology and natural history of pertussis, and the difficulty encountered by mathematical modelers in building well calibrated simulations for this disease imply that substantial gaps in knowledge still exist [43]. We evaluated the epidemiology of pertussis over a 14-year period in a large urban-suburban region of Ontario, Canada. In the Greater Toronto Area, pertussis incidence was approximately stable in the early 1990s, increased somewhat with the introduction of polymerase chain reaction (PCR) in 1999, and then increased markedly with the introduction of a new, highly sensitive PCR assay. Because this was a laboratory-derived, population-based analysis (due to the fact that all pertussis testing in the region is performed by the two laboratories participating in this study), we had the opportunity to evaluate both changes in disease epidemiology and changes in testing volumes and test technology, and to examine the interplay between these two distinct processes.

We found that the surge in pertussis incidence in the GTA from 2005 to 2007 likely reflected a combination of factors, with a true, underlying increase in disease risk coinciding with the introduction of a highly sensitive assay that probably identified cases that would have been missed with earlier testing modalities. Surges in apparent disease incidence with the introduction of highly sensitive assays for pertussis have been described in other settings [44], but the process we identify here is not as simple as increasing case counts as a result of increased test sensitivity. Instead, through the use of time series analysis, we were able to identify positive feedback loops, whereby increasing test positivity (due to either increasing incidence or increased test sensitivity) led to increased test submissions by clinicians over subsequent weeks. Such positive feedbacks were previously noted by us in the context of legionellosis [45] but to our knowledge this is the first time such feedbacks have been described for pertussis.

The ability to control for both test volumes and testing technology provides a more nuanced view of the state of pertussis epidemiology in this jurisdiction than would be possible using usual surveillance data sources, which focus on "positive" cases alone, and do not consider frequency of testing. In this instance, adjustment for test frequency reduced the relative risk of pertussis in the youngest children, and also diminished the apparent impact of the introduction of a highly sensitive PCR assay on pertussis incidence. Unfortunately, few jurisdictions have the ability to adjust for testing practices and volumes when analyzing disease trends, but our findings show that these factors may be important, particularly when real or artifactual surges in disease create public concern, leading to further increases in testing. This is a concept which has application beyond pertussis; for example, Vickers and Osgood [46] evaluated an increase in Chlamydia incidence in the Canadian province of Saskatchewan which has centralized testing such that test volumes are available; in a mathematical model, they found that changes in test volume alone, rather than changes in testing characteristics or true underlying Chlamydia risk, were likely responsible for the surge in observed Chlamydia incidence in that province. These authors noted "diminishing returns" from increasing test submissions, as the marginal identification of cases declines with expansion of testing. Statistical evidence for such a phenomenon is seen in our analysis too, in the diminished (though still statistically significant) association between test submission volumes and estimated relative risk from May 2005 onwards.

We identified other important changes in the apparent epidemiology that appear to be driven by testing practices rather than disease activity per se. For example, using spectral decomposition, we found that whereas pertussis in the GTA displays (expected and typical) autumn seasonality, but pertussis testing displays wintertime seasonality (perhaps due to the use of pertussis testing in individuals with undifferentiated cough illness due to wintertime respiratory viruses). This might be expected to influence observed seasonal patterns of disease occurrence, and again confirms the degree to which a nuanced understanding of disease epidemiology requires some knowledge of how information on disease trends is obtained at the level of the microbiology laboratory.

Like any epidemiological analysis, our analysis is subject to limitations. Key among these relates to measurement. Although an attempt was made to describe trends in pertussis in a population with centralized testing, it is unlikely that all individuals with pertussis underwent testing, and inasmuch as both likelihood of symptoms and likelihood of testing depend on age, some of the effects reported here may relate to differential testing of different groups. This is a limitation of any population-based study of pertussis epidemiology. In addition, we had no access to information on individuals with clinical pertussis without laboratory testing, though given universal healthcare coverage in this region and the ready availability of laboratory tests, it is unlikely to have had a major influence on key findings.

## Conclusion

In summary, the epidemiology of pertussis in a large urban-suburban region of Ontario, Canada with centralized pertussis testing over a 15-year period identified surges in pertussis incidence in this jurisdiction from 2005 onwards. The ability to control for laboratory test technology and test volume allowed identification of numerous components to this rise in incidence, including improved test sensitivity and a previously undescribed (for pertussis) "positive feedback loop" whereby increasing reported disease activity appeared to cause subsequent surges in laboratory test submissions by clinicians. We were also able to detect distinctive seasonality of pertussis identified by PCR testing without culture confirmation. In conclusion, the microbiology laboratory is a key component of smart, integrated disease surveillance infrastructure; where possible, trend estimates for pertussis and other communicable diseases should incorporate data on laboratory test volumes and technologies, in order to inform smarter and more efficient public health policy.

## Competing interests

DNF has received unrestricted research and/or educational funding from Sanofi Pasteur, Novartis, and GlaxoSmithKline, all of which are manufacturers of vaccines, including vaccines against pertussis and other respiratory infections. All other authors declare that they have no competing interests.

## Authors' contributions

DNF helped design the study, performed statistical analyses of epidemiological data, co-wrote the initial draft of the manuscript, and revised the manuscript for important intellectual content. PT created, formatted, and maintained the main pertussis database used for this study and contributed to critical revision of the manuscript. TH contributed to statistical analyses, co-wrote the initial draft of the manuscript and contributed to critical revision of the manuscript. SR obtained laboratory from the Hospital for Sick Children and contributed to interpretation of study results and critical revision of the manuscript. DEL contributed to interpretation of study results and critical revision of the manuscript. FJ helped design the study, and contributed to interpretation of study results and critical revision of the manuscript. All authors read and approved the final version of the manuscript.

## Pre-publication history

The pre-publication history for this paper can be accessed here:

http://www.biomedcentral.com/1471-2458/11/694/prepub
